# Benign Vomiting-Induced Hepatic Portal Venous Gas: A Case Report

**DOI:** 10.7759/cureus.82512

**Published:** 2025-04-18

**Authors:** Zann Der Yeo, Riaz Dor

**Affiliations:** 1 Gastroenterology, Queen's Hospital Burton, University Hospital Derby and Burton NHS Foundation Trust, Burton-on-Trent, GBR

**Keywords:** diabetic gastroparesis (dg), hepatic portal vein gas, nausea and vomiting, radiological, radiological findings, starvation ketosis, vomitting

## Abstract

Hepatic portal venous gas (HPVG) is a rare but significant radiological finding traditionally associated with severe abdominal pathology, particularly bowel ischaemia. However, advances in imaging have led to the recognition of benign and self-limiting causes. We report the case of a 34-year-old female with longstanding type 1 diabetes mellitus, autonomic dysfunction, and stage 4 chronic kidney disease (CKD), who presented with severe vomiting, abdominal pain, and malaise. CT revealed HPVG without any evidence of bowel compromise or ischaemia. The patient was successfully managed with conservative treatment, including intravenous fluids, antiemetics, and insulin therapy, resulting in rapid clinical improvement and resolution of the HPVG on follow-up imaging. This report underscores the importance of recognising vomiting-induced HPVG as a benign phenomenon and highlights the essential role of careful clinical assessment in avoiding unnecessary surgical interventions.

## Introduction

Hepatic portal venous gas (HPVG) refers to the presence of gas within the portal venous system, typically seen on CT as tubular areas of decreased attenuation in the liver [1]. It is recognised as a radiological finding rather than a diagnosis itself. First described by Wolfe and Evans in 1955 in neonates with necrotising enterocolitis [2], HPVG was historically linked to severe bowel ischaemia, carrying mortality rates as high as 75% [3]. However, increasing availability and sensitivity of advanced imaging have led to greater detection of HPVG in various benign and self-limiting conditions, highlighting a broader spectrum of underlying causes. Despite this progress, distinguishing between ischaemic and non-ischaemic HPVG remains challenging, partly due to the absence of clear, evidence-based management guidelines [4].

We present a case of vomiting-induced HPVG in a clinically stable patient with type 1 diabetes mellitus and chronic kidney disease (CKD), successfully managed with supportive treatment. This report emphasises the importance of careful clinical risk stratification to avoid unnecessary surgical interventions.

## Case presentation

A 34-year-old female with type 1 diabetes mellitus (diagnosed in 2006), chronic axonal motor sensory neuropathy, stage 4 CKD (CKD4; awaiting transplant evaluation), and autonomic dysfunction presented to the emergency department with severe vomiting, abdominal pain, and malaise lasting 24 hours. She reported vomiting food contents, without melaena, diarrhoea, or urinary symptoms. On arrival, she was found to be hypotensive (BP: 98/67 mmHg), tachycardic (HR: 112 bpm), and tachypnoeic (RR 20/min), with a normal temperature. Examination revealed epigastric tenderness without features of peritonism. Given her history of poor oral intake and metabolic stress, a provisional diagnosis of starvation ketoacidosis with possible Mallory-Weiss tear was considered.

Initial blood tests demonstrated mild leucocytosis, likely reflecting a physiological stress response, and normocytic anaemia, consistent with anaemia of chronic disease in the context of longstanding CKD. Capillary ketones were elevated, supporting the provisional diagnosis of starvation ketosis. Renal function was in keeping with her baseline CKD4 (Table 1). Venous blood gas analysis revealed a normal pH and lactate, with a mildly elevated bicarbonate and base excess, suggestive of early metabolic alkalosis likely secondary to repeated vomiting and volume depletion (Table 2).

**Table 1:** Blood test analysis revealing abnormal white cell count, haemoglobin, ketones, urea, creatinine, and eGFR CRP: C-reactive protein; eGFR: estimated glomerular filtration rate

Blood test	Patient value	Units	Range
White Cell Count	12.6	10^9^/L	4.0 – 11.0
Haemoglobin	115	g/L	120 – 150
CRP	<5	mg/L	0.0 – 5.0
Sodium	141	mmol/L	133.0 – 146.0
Potassium	4.1	mmol/L	3.5 – 5.3
Ketones	1.9	mmol/L	<0.6
Urea	11.8	mmol/L	2.5 – 7.8
Creatinine	159.0	micromol/L	45.0 – 84.0
eGFR	36.3	ml/min	90.0 – 120.0

**Table 2:** Venous blood gas analysis demonstrating mildly elevated bicarbonate and base excess, normal lactate BE: base excess; HCO₃⁻: bicarbonate; VBG: venous blood gas

VBG parameter	Results	Units	Reference range
pH	7.42	-	7.35 – 7.45
HCO₃⁻	27.6	mmol/L	22 – 26
BE	+4.1	mmol/L	-2 – +2
Lactate	1.0	mmol/L	0.5 – 2.2

**Figure 1:**
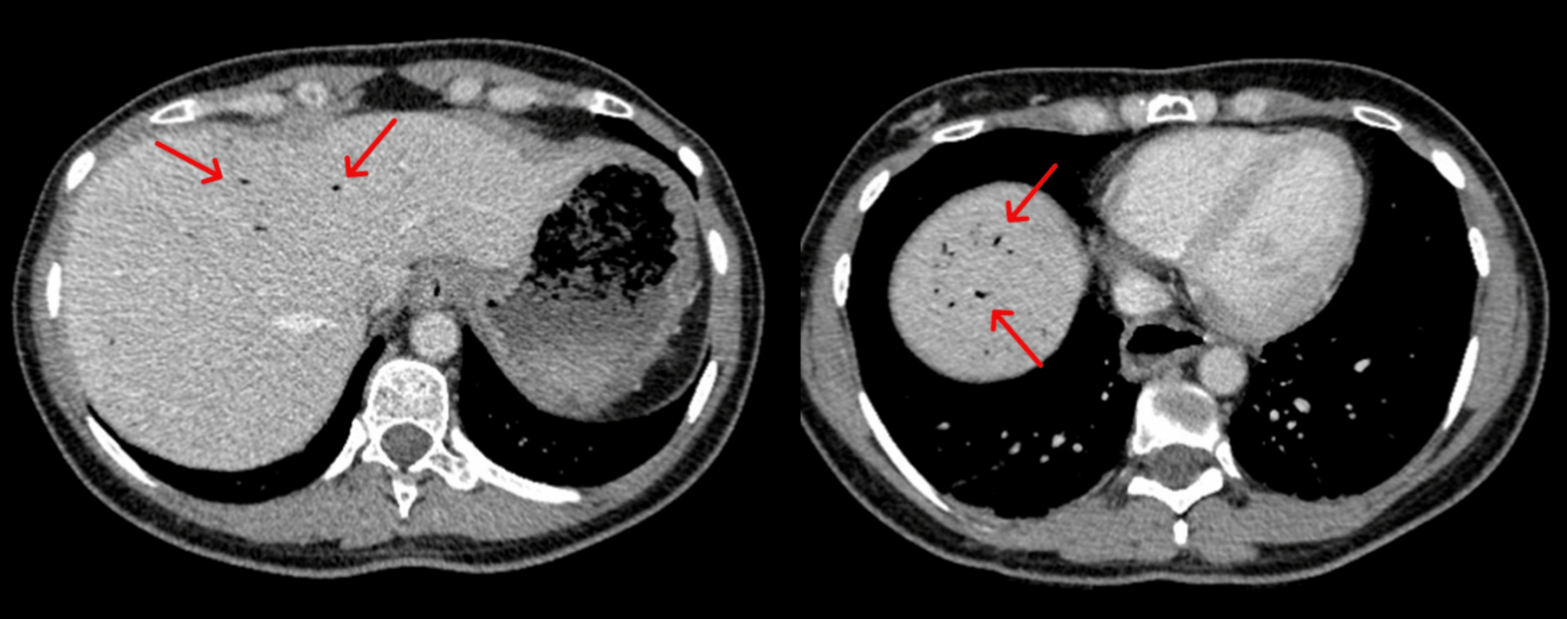
Transverse CT image showing marked locules of hepatic portal venous gas CT: computed tomography

**Figure 2:**
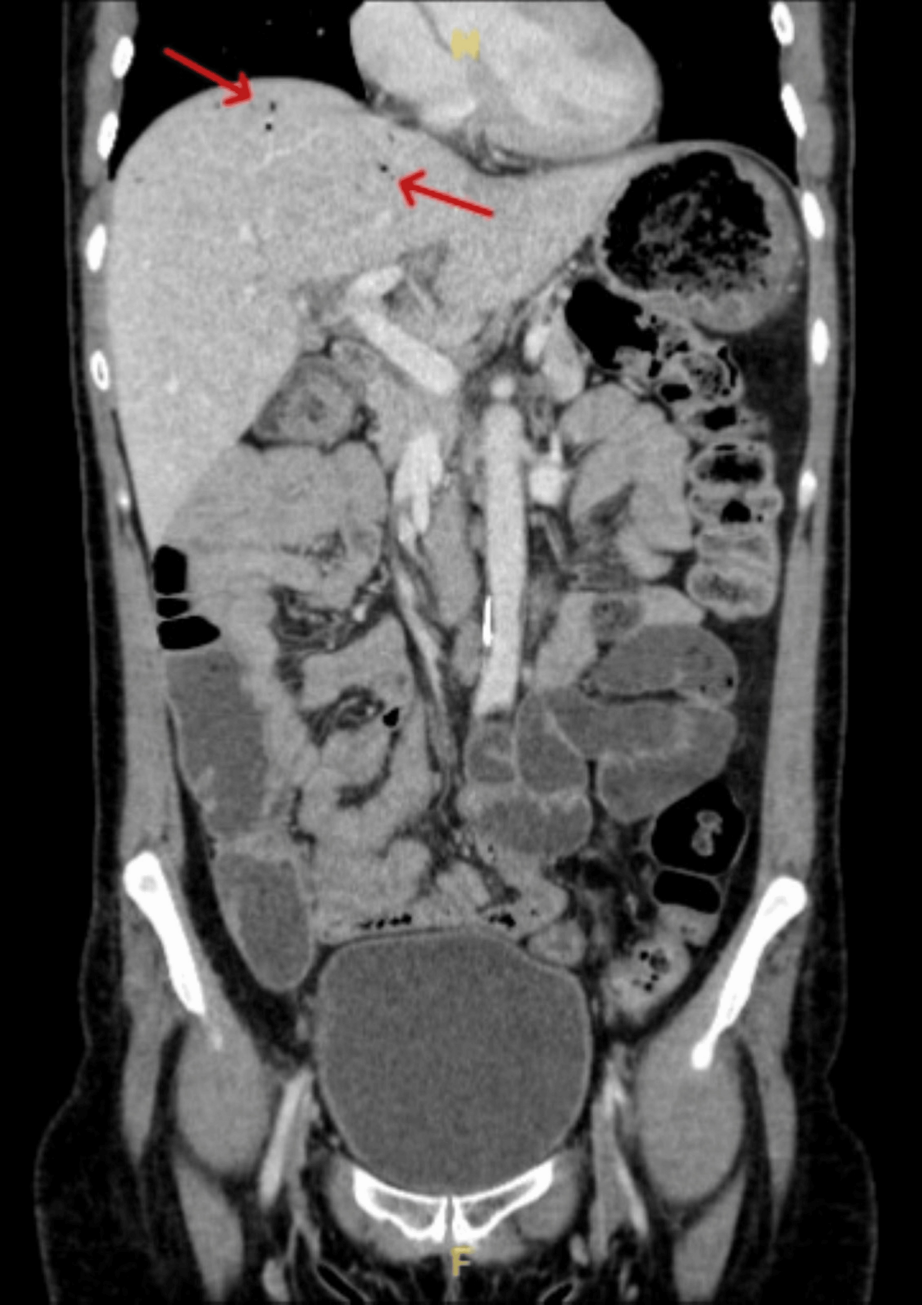
Coronal CT image demonstrating marked portal venous gas distributed throughout the periphery of the liver No pneumatosis intestinalis, bowel wall thickening, or intra-abdominal free air are seen. The remainder of the abdomen, including unprepared bowel loops, appears unremarkable CT: computed tomography

A CT abdomen and pelvis with contrast was performed, which revealed multiple hepatic gas locules consistent with HPVG, but there was no evidence of bowel ischaemia, pneumatosis intestinalis, or perforation (Figures 1, 2). The portal vein was patent, and no evidence of intra-abdominal sepsis was identified.

**Figure 3:**
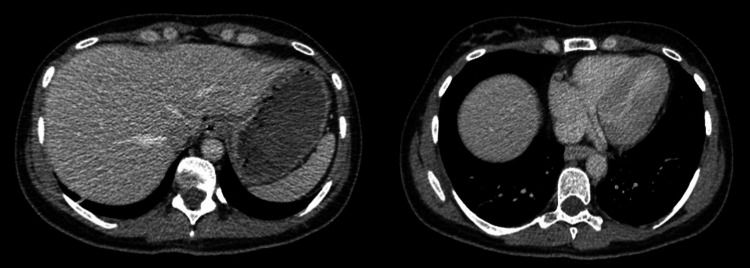
Follow-up CT showing complete resolution of HPVG CT: computed tomography; HPVG: hepatic portal venous gas

Despite the significant radiological findings, the patient was clinically and haemodynamically stable. While bowel ischaemia remained the most critical differential diagnosis, this patient’s normal lactate, absence of bowel thickening or pneumatosis on CT, and rapid clinical improvement with conservative measures suggested a benign aetiology. A conservative approach was adopted, including intravenous hydration, antiemetics, and glucose control with variable rate insulin infusion (VRII). The patient's condition significantly improved over the next 48 hours. Repeat imaging on day three showed complete resolution of HPVG (Figure 3), and the patient was transitioned back to subcutaneous insulin once oral intake had improved. She was discharged with follow-up in the outpatient diabetes clinic.

## Discussion

While HPVG is historically associated with mesenteric ischaemia, its presence is now increasingly recognised in a range of non-ischaemic and benign conditions. These include vomiting-induced mucosal injury, bacterial translocation in cases of enteritis or infection, and iatrogenic causes such as endoscopic procedures or feeding tube insertion [5-7]. In some patients, HPVG has even been reported without any identifiable underlying pathology, challenging the traditional assumption that it always signifies a surgical emergency [6]. Recognising this wide range of aetiologies reinforces the need for a broad differential diagnosis when evaluating HPVG, especially in clinically stable patients.

Distinguishing between ischaemic and benign HPVG is vital, as the treatment approach varies significantly. Ischaemic HPVG typically presents with elevated lactate levels, metabolic acidosis, and imaging features such as bowel wall thickening or pneumatosis intestinalis, suggestive of infarction or necrosis [7,8]. In contrast, benign HPVG is usually seen in clinically stable patients with normal lactate levels and unremarkable bowel findings on CT [7,8]. Importantly, some patients with early ischaemia may initially appear stable, and hence serial assessments and clinical vigilance remain crucial [3,8].

In our case, the likely cause of HPVG was vomiting-induced gastric mucosal injury, leading to increased mucosal permeability, allowing the intraluminal gas to enter damaged mucosa and track into the portal venous system [7]. Diabetic gastroparesis and autonomic dysfunction may have exacerbated gastric stasis and distension, increasing mucosal vulnerability. A similar case involving a critically ill patient with longstanding diabetes mellitus and features of gastroparesis was also managed conservatively without surgical intervention, further demonstrating that vomiting-induced HPVG can be resolved with supportive care alone [9].

Ultimately, clinical risk stratification should guide decision-making. The absence of acidosis, haemodynamic compromise, or bowel wall changes supported a conservative approach in our patient, who responded well to supportive care. Repeat imaging confirmed resolution of the HPVG, emphasising that not all cases necessitate surgical intervention and highlighting the importance of integrated clinical and radiological assessment.

## Conclusions

This report demonstrates that HPVG, while often considered a radiological marker of life-threatening pathology, can also arise in benign settings such as vomiting-induced mucosal injury. Recognising this distinction is crucial. A thorough clinical assessment -particularly evaluating haemodynamic stability, lactate levels, and radiological findings - can help differentiate between ischaemic and non-ischaemic causes. In appropriately selected patients, a conservative approach can lead to complete resolution, thereby sparing them from unnecessary interventions.
